# Quantitative analysis of fine dust particles on moss surfaces under laboratory conditions using the example of *Brachythecium rutabulum*

**DOI:** 10.1007/s11356-021-14218-5

**Published:** 2021-05-15

**Authors:** Bilitis Désirée Vanicela, Martin Nebel, Marielle Stephan, Christoph Riethmüller, Götz Theo Gresser

**Affiliations:** 1 German Institutes of Textile and Fiber Research, Körschtalstraße 26, 73770 Denkendorf, Germany; 2 University of Bonn, Regina-Pacis-Weg 3, D-53113 Bonn, Germany

**Keywords:** Moss, Bryophytes, *Brachythecium rutabulum*, Particle counting, Fine dust particle, SEM

## Abstract

The identification of a model organism for investigations of fine dust deposits on moss leaflets was presented. An optical method with SEM enabled the quantitative detection of fine dust particles in two orders of magnitude. Selection criteria were developed with which further moss species can be identified in order to quantify the number of fine dust particles on moss surfaces using the presented method. Among the five moss species examined, *B. rutabulum* had proven to be the most suitable model organism for the method presented here. The number of fine dust particles on the moss surface *of B. rutabulum* was documented during 4 weeks of cultivation in the laboratory using SEM images and a counting method. The fine dust particles were recorded in the order of 10 μm–0.3 μm, divided into two size classes and counted. Under laboratory conditions, the number of particles of the fine fraction 2.4 μm–0.3 μm decreased significantly.

## Introduction

Particulate matter (PM) is a contributing factor, responsible for up to 45,000 deaths per year in Germany (Umweltbundesamt 2018). It describes all particles smaller than 10.0 μm. The particulate matter is divided into fractions, according to the size of the particles: the “coarse fraction” (10.0 μm > PM 10 >2.5 μm), the “fine fraction” (2.5 μm > PM 2.5 > 0.1 μm), and the “ultrafine fraction” (< 0.1 μm) (Umweltbundesamt 2018). The main sources of particulate matter are traffic (exhaust gases, tire, brake, and road surface abrasion), heating, industrial emissions and agriculture (Umweltbundesamt 2018). The measurement method in Germany is carried out via so-called active collectors, according to the standard method established by the European Committee for Standardisation (CEM). The outside air is passed through a size-selective sample inlet at a known constant flow rate. The PM fraction of interest is collected on a filter for a known nominal duration of 24 h. The mass of the PM material is determined by weighing the filter before and after collection of the dust under predetermined constant conditions (DIN EN 12341 2014). The measuring stations in urban environments collect only data about PM 10 and PM 2.5 without further differentiation. Data about the weight and not about the number of particles is recorded (Stadtklima-Stuttgart 2020). The limit value recommended by the WHO only takes into account the weight and not the number of particles per volume (Umweltbundesamt 2020a, 2020b, 2020c). Although the weight of the small fraction and ultra-fine fraction particles is much lower, the total number of particles is much higher compared to the particles of the coarse fraction (Tittarelli et al. 2008). The fine and ultra-fine particles in particular are responsible for the health consequences of particulate pollution (Spektrum.de 2020). The available scientific data is still very incomplete today.

Particulate matter is deposited on the surfaces of plants, mainly on the leaves (Zheng and Li 2019; Kappis et al. 2007). Mosses, unlike flowering plants, have only a very thin layer of wax (cuticle) on the surface of their leaflets; therefore, they are able to absorb water and nutrients over the whole surface area. They are more exposed to environmental influences on account of their very thin cuticle and have therefore been used as biomonitors for air quality for over 40 years (Ares et al. 2012). Spagnuolo et al. (2011) showed that mosses have a better ability to intercept and accumulate airborne elements compared to other biomonitors. Adamo et al. (2007) suggested surface structures of mosses to explain this observation. This simple and efficient method of moss bag monitoring which was widely used allowed the monitoring of a large number of pollutants. However, there is a lack of uniform standard protocols for moss bag monitoring (Ares et al. 2012). Besides the problem of missing standard protocols, the elements found in the moss samples are the result of additive uptake and also the loss of accumulated elements through leaching. Therefore, it is not possible to establish a linear relationship with the atmospheric deposition values (Ares et al. 2012).

A lot of publications show that extensive data sets were collected and evaluated, which show a relationship between the atmospheric concentration of numerous heavy metals and other chemical compounds and the corresponding concentration in moss samples. Some of the samples were collected and examined in high numbers across Europe (Schröder et al. 2010a, 2010b; Harmens et al. 2010, 2011, 2012). The binding and uptake of fine dust particles is facilitated by their high ion exchange capacity (Frahm 2001; Fabian et al. 2011; Maxhuni et al. 2015). While the penetration mechanism remains unknown, Canivet et al. (2014) demonstrated the uptake of iron nanoparticle agglomeration as mineral water suspension with *Aphanorrhegma patens*. The particles were detected inside the cell.

Observations of particulate matter deposited on moss leaflets have seldom been presented using SEM micrographs, until now (Hofman et al. 2017; Di Palma et al. 2017; Tretiach et al. 2011; Weinbruch 2010). Studies on *Brachythecium rutabulum* show a high number of fine dust particles on the leaflets (Nebel, unpubl.).

Di Palma and colleagues investigated particulate matter deposition on *Pseudoscleropodium purum* at rural, urban, and industrial sites at three locations in Europe. The samples were examined using SEM/EDX and evaluated, among other factors, with regard to the number and size of particles (Di Palma et al. 2017). Tretiach et al. (2011) investigated particulate matter with respect to the composition and distribution of size fractions. This study includes particle counts on moss surfaces for a small random sample size.

The aim of this paper is to present a method for the selection of a suitable moss and the quantitative analysis of the account of particulate matter on its leaflet surfaces.

## Methods

### Collection sites of the examined mosses

**Fig. 1:**
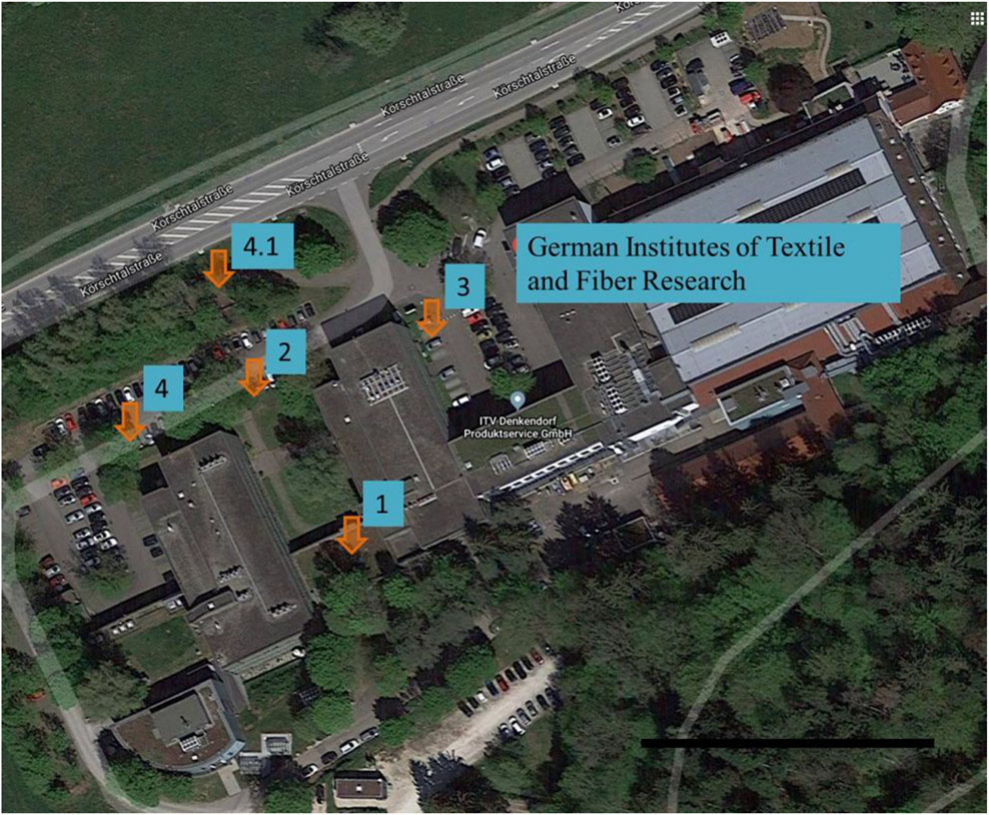
Locations of sample collection: (1) *C. purpureus*, (2) *H. cupressiforme*, (3) *S. convolutum*, and (4) *B. rutabulum* for part 1 and (4.1) *B. rutabulum* for part 2; the map was derived from Google Maps and edited with MS Power Point, scale: 50 m. A *B. rutabulum* moss cushion was taken from a tree trunk at 0.2-m height and distance of 21 m of a busy country road (see Fig. 2). It was on the side of the tree trunk facing the country road in a northwesterly direction

Five mosses were taken from nature and examined. The mosses were all collected on the site of the German Institutes of Textile and Fiber Research in Denkendorf (see Fig. 1) except for *R. canescens*, which was purchased commercially and had been cultivated in Kirchzarten, Germany.

**Fig. 2:**
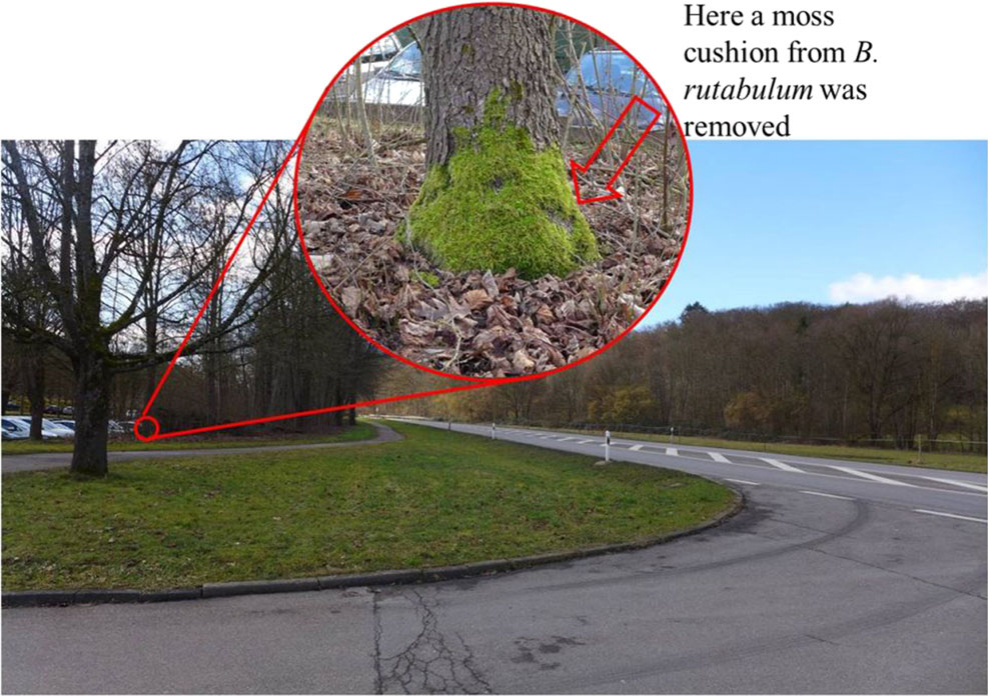
Collection site of *B. rutabulum.* The photo was edited with MS Power Point

### Examination of particulate matter deposits on moss surface

In preparation for the investigation with the scanning electron microscope (SEM), moss samples were taken turgescent directly from the cultivation status in the laboratory and the individual leaflets removed with tweezers, as completely as possible (Dumont No. 5) and stuck onto a sample plate. This preparation was carried out under a binocular microscope (Wild, photomacroscope M400). The moss leaflets were alternately fixed with the top and underside facing up. After drying, they were coated with gold palladium (Balzers Union, SCD 040, sputtering time 40 s). Images of the moss leaflets were taken with the scanning electron microscope (Hitachi Tabletop Microscope TM-1000). A position in the middle of the leaf blade was chosen to record the image and an image was taken of each moss leaflet at 2000- and 3000-fold magnification.

### Cultivation of *B. rutabulum* in the laboratory

**Fig. 3:**
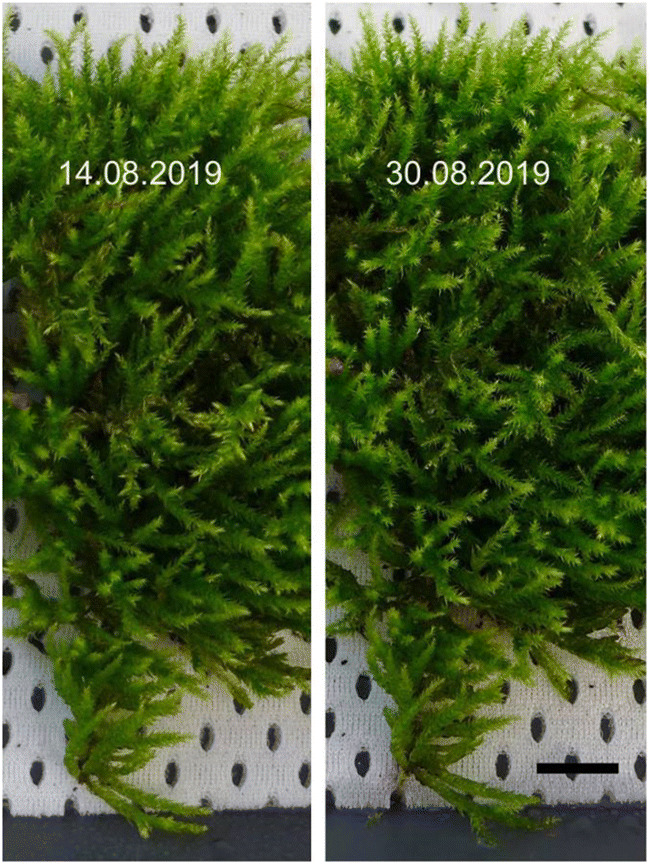
Extract from the photo documentation of the cultivation of *B. rutabulum* in the laboratory, scale: 2 cm

The moss cushion was applied to a textile surface under laboratory conditions (23 °C ± 1 °C, 43% ± 5% humidity, ventilation 550 m^3^/h, ventilation system with filter class F9) and moistened from above every 2 to 3 h, for 6 to 8 s, using very finely atomized demineralized water. According to EMW (2021), the ventilation system used here filters out particles of the size range considered here with an average efficiency of 95% and a minimum efficiency of 70%. As a reference, moss leaves grown in the laboratory were examined for particles (PM 10 and PM 2.5) on their surface (see Table 5). The growth of the moss plants was photographed during the course of the experiment (see Fig. 3). SEM samples were taken on the day of collection and after 2, 7, 14, 21, and 28 days of cultivation under laboratory conditions. The sample size varies between 15 and 48 for the coarse fraction and 12–15 for the fine fraction (see Tables 3 and 4). The multi-stage sample preparation process has as a consequence that the actual sample size of the respective sampling is only known with a time delay.

### Counting of particulate matter particles on surfaces of *B. rutabulum* in two orders of magnitude

The number of particles was determined as follows. The quantitative evaluation was carried out manually using an image processing program (analySIS Digital Image Analysis, Olympus Soft Imaging Solutions GmbH). The particles of the respective fraction were marked manually and the number recorded by the image processing program. The coarse and fine fractions were assessed separately.

To quantify the coarse fraction, a grid with an edge length of 2.5 μm was placed over the SEM image. All particles larger than the spaces created by the grid were counted. The image field was set to 100 × 100 μm.

**Fig. 4:**
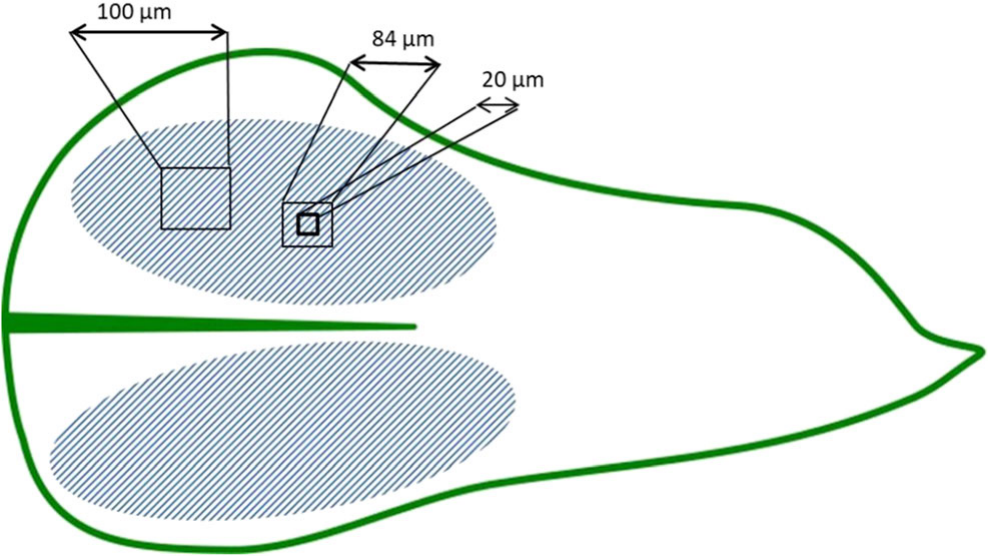
Schematic representation of the counted area. The SEM images were always taken in the area of the leaf blades (hatched area here). The SEM images for counting the coarse particles are 100 × 100 μm. The SEM images for counting the fine particles were 84 × 84 μm; here the counted area was further limited to 20 × 20 μm (square with thick frame line). The graphic was designed with inkscape and MS Power Point

**Fig. 5:**
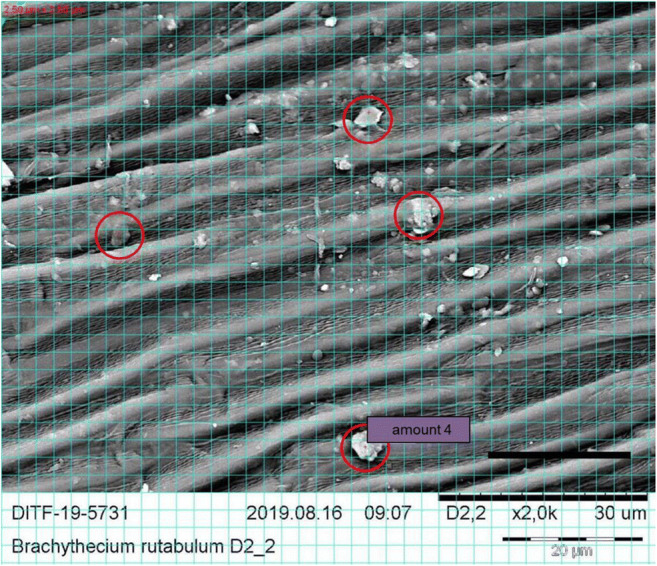
SEM image of 2000 times magnification of *B. rutabulum*. The grid with an edge length of 2.5 μm is shown in light blue. The coarse particles counted are marked with a red marking. Scale: 20 μm. The photo was edited with MS Power Point

**Fig. 6:**
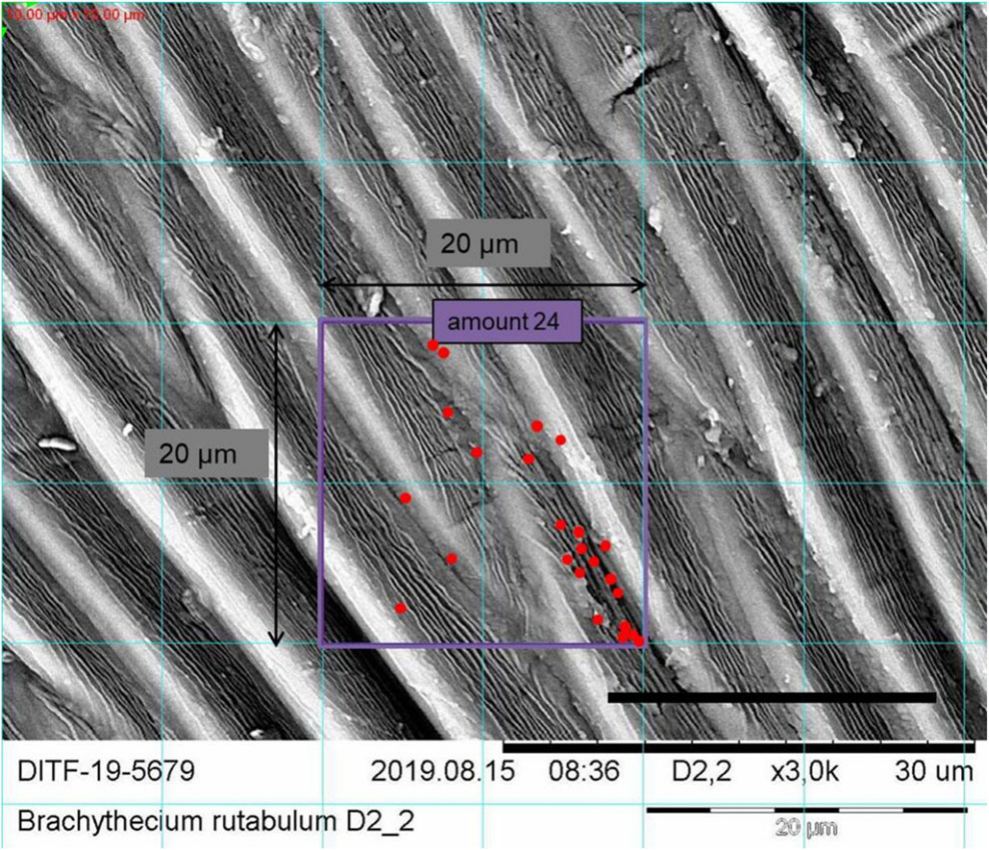
SEM image of ×3000 magnification of *B. rutabulum*. The area marked in purple shows a 20 × 20 μm area that was counted. The counted fine particles marked with red. Scale: 20 μm. The photo was edited with MS Power Point

To count the fine fraction, a grid with edge length of 10 μm was used to delimit the field in the SEM image of 84 × 84 μm in order to find a 20 × 20 μm field in which the moss surface is sharply defined and the fine particles could be counted correctly (see Figs. 4, 5, and 6).

The results of the count were examined statistically. Single factor variance analysis (ANOVA) was done with Microsoft Office Excel 2010.

**Fig. 7:**
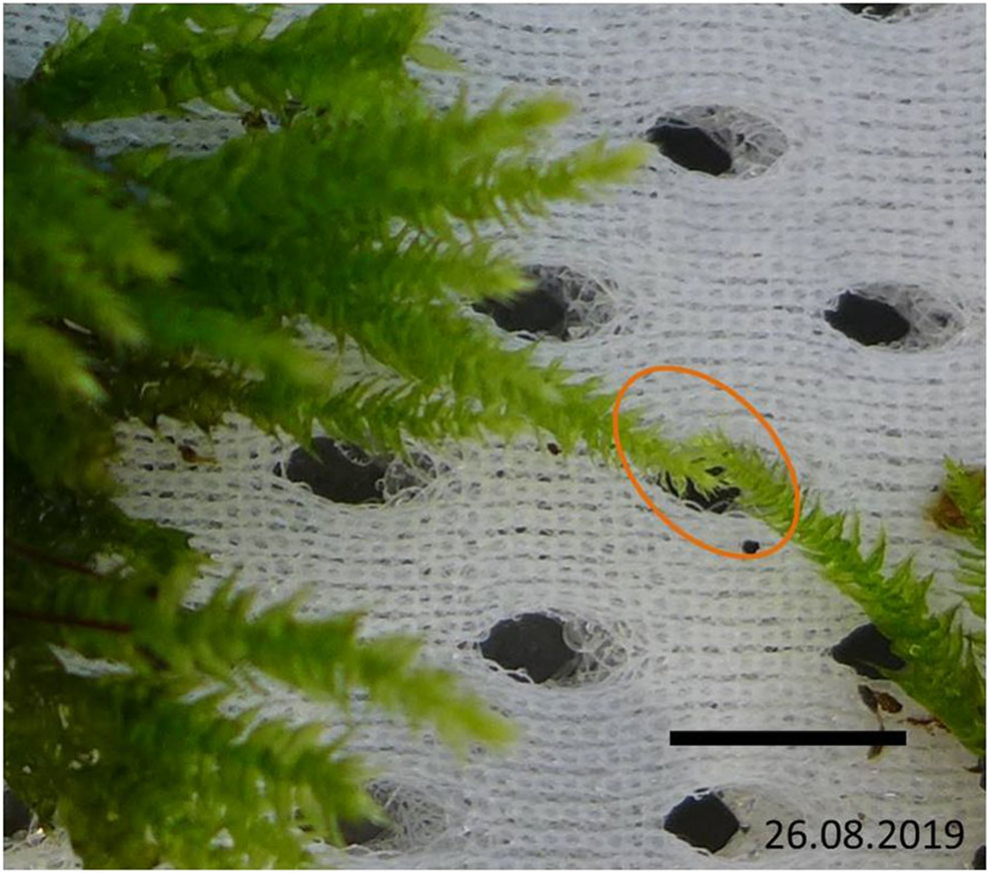
The marked area shows the newly grown leaves in the laboratory. They differ from the existing leaves in that they are significantly smaller and lighter. These sheets were examined to determine the basic load in the laboratory while the experiment was being carried out. Scale: 1 cm. The photo was edited with MS Power Point

In order to record the basic load with particulate matter in the laboratory during the experiment, newly grown leaflets were removed from the moss cushion in the laboratory and subjected to the same procedure (see Fig. 7). Samples were taken on cultivation day 14, 21, and 28.

## Results

### Moss selection

**Fig. 8:**
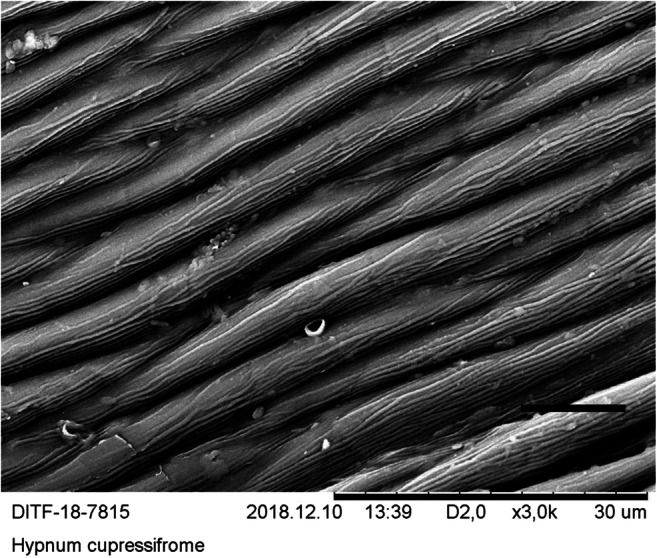
SEM images, 3000-fold from *C*. *purpureus*, scale: 10 μm

**Fig. 9:**
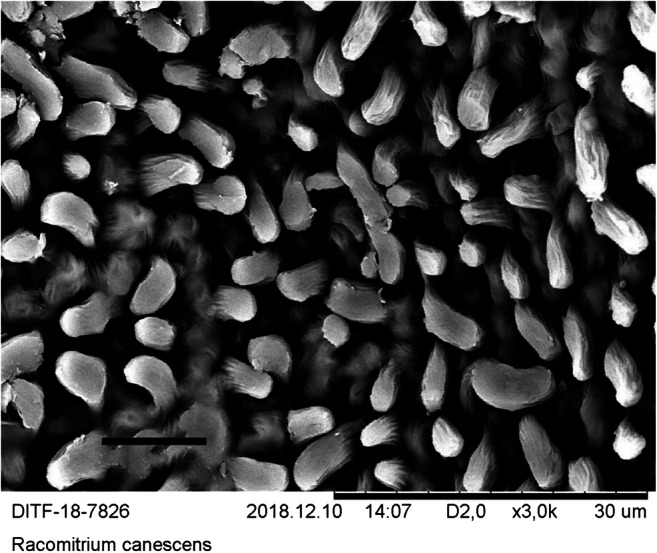
SEM images, 3000-fold from *S. convolutum*, scale: 10 μm

**Fig. 10:**
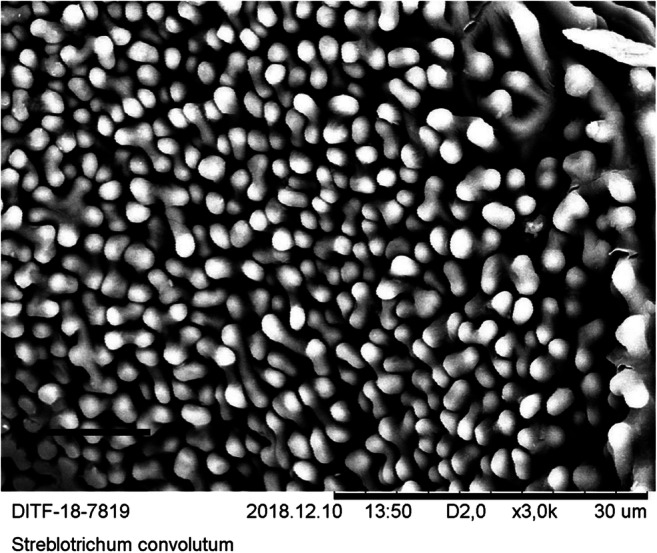
SEM images, 3000-fold from *R. canescens*, scale: 10 μm

**Fig 11:**
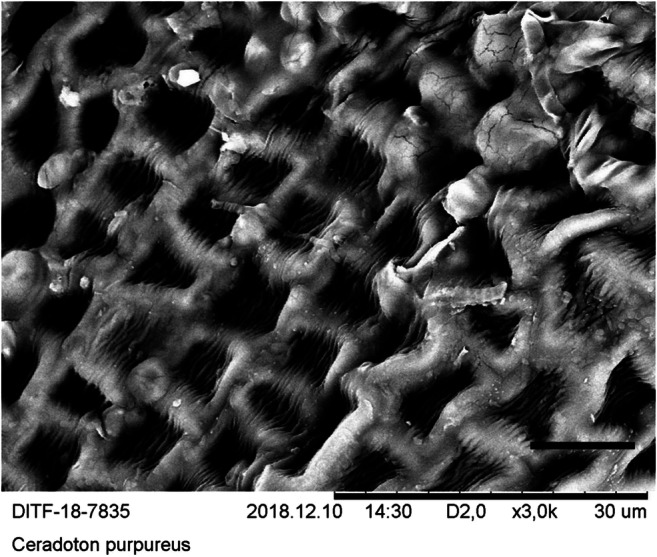
SEM images, 3000-fold from *H. cupressiforme*, scale: 10 μm

**Fig. 12:**
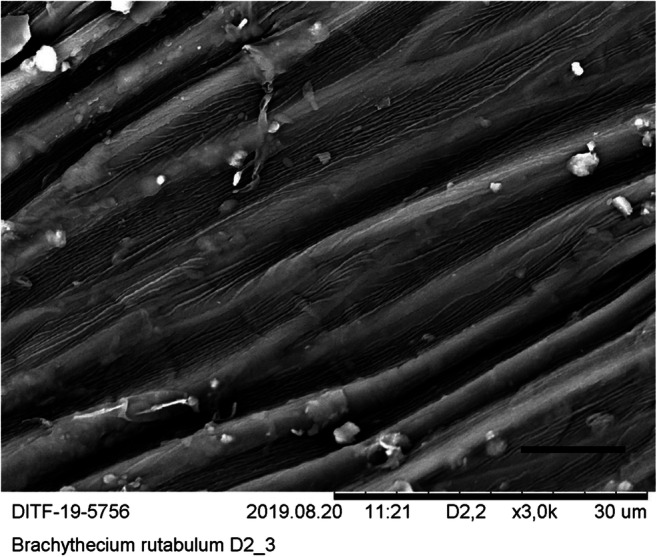
SEM images, 3000-fold *B. rutabulum*, scale: 10 μm

Since the quantification of the particles is time-consuming and expensive, a suitable moss species must be identified before the quantification procedure can be carried out. For this purpose, five moss species were identified with regard to the criteria surface condition, preparation of the sample for microscopy, visibility, and frequency of the particles on the surface. In the bottom row of Tables 1 and 2, the selection procedure was evaluated in terms of suitability for the method. SEM micrographs were created of the surfaces of these species (see Figs. 8, 9, 10, 11, and 12). The following species were investigated: *Ceratodon purpureus*, *Streblotrichum convolutum*, *Racomitrium canescens*, *Hypnum cupressiforme*, *Brachythecium rutabulum.*

**Table 1:** Moss selection

Species	*C. purpureus*	*S. convolutum*	*R. canescens*
Surface	Cells quadrate, smooth, with small lumen and thick walls, leaves small, with recurved margin	Cells small, rounded, with papillae, very small leaves with recurved margin	Cells elongate quadrate, with numerous large papillae, medium sized leaves with recurved margin
Preparation	Difficult	Difficult	Easy
Particles	Clearly visible, few	Particles are difficult to detect due to papillae	Particles are difficult to detect due to papillae
SEM image	Fig. 8	Fig. 9	Fig. 10
Suitability for presented method	**→**moderate suited	**→** not suited	**→** not suited

**Table 2:** Moss selection

Species	*H. cupressiforme*	*B. rutabulum*
Surface	Cells elongate, narrow, smooth, leaves medium sized, concave, weakly falcate	Cells elongate, narrow, smooth, leaves medium sized to large, smooth to slightly plicate
Preparation	Easy	Easy
Particles	Clearly visible, moderate number	Clearly visible, numerous
SEM image	Fig. 11	Fig. 12
Suitability for presented method	→ suited	→ best suited

The selection process has shown that *B. rutabulum* (see Fig. 12) is best suited as a model organism for the method presented here. Based on the results shown in Tables 1 and 2, the investigations to quantify the fine dust particles were carried out for *B. rutabulum*. In particular, the most important point for the method, namely the visibility of the particles on the moss surface, justifies this approach.

### Quantification of particulate matter in two orders of magnitude

**Fig. 13:**
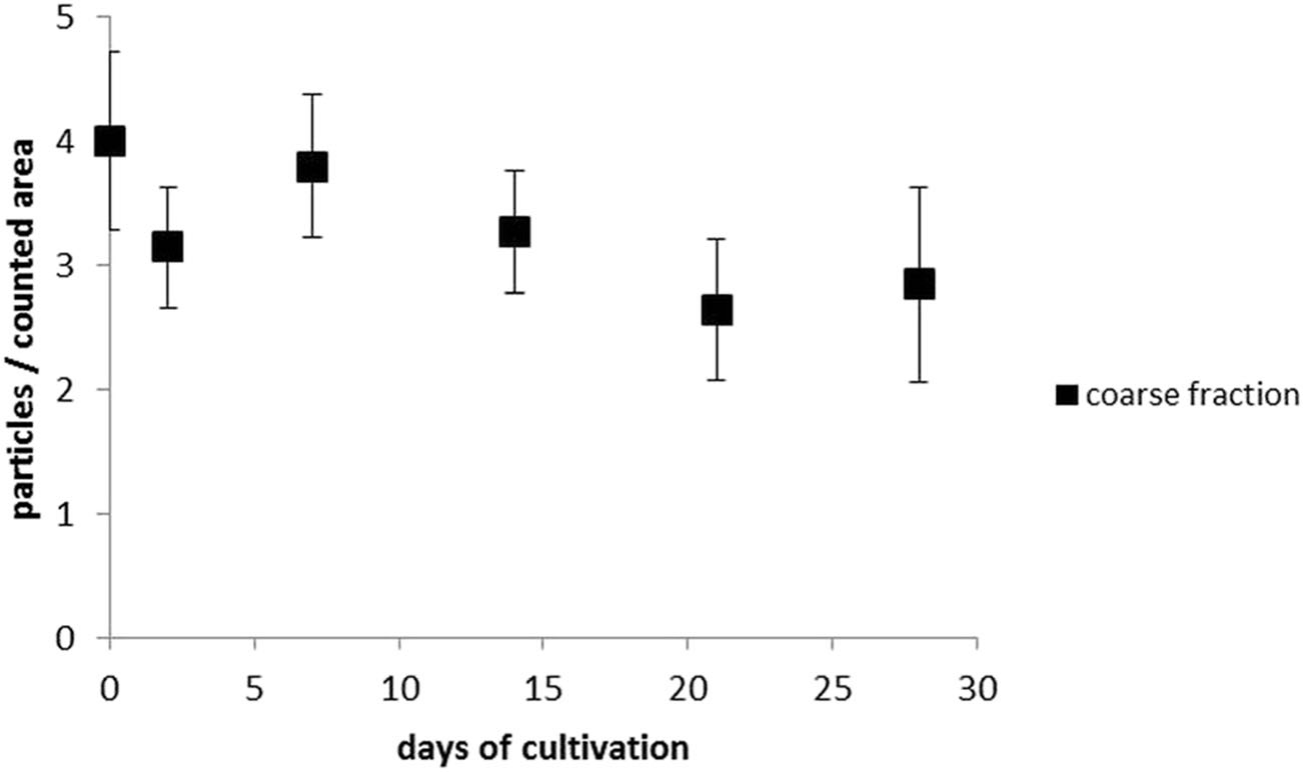
The number of coarse particles of the counted area (100 × 100 μm) is plotted over the cultivation period. The markings show the mean with the standard error of the mean plotted up and down. The graphic was created with MS Excel

**Fig. 14:**
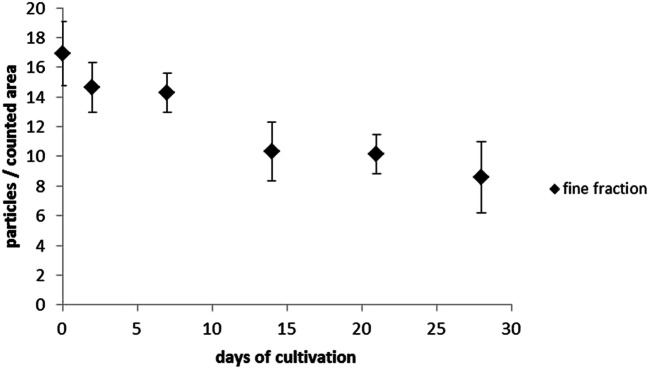
The number of fine particles of the counted area (20 × 20 μm) is plotted over the cultivation period. The markings show the mean with the standard error of the mean plotted up and down. The graphic was created with MS Excel

The results of the quantitative analysis of the particles are shown in Figs. 13 and 14. The results of the coarse and fine particles were shown separately. The sample size showed the number of counted leaflets (see Tables 3 and 4).

**Table 3:** Sample sizes of the quantitative analysis of the coarse particles (2.5 μm–10.0 μm)

Days of cultivation	Start	2	7	14	21	28
Sample size (*n*)	23	21	48	15	25	20

**Table 4:** Sample sizes of the quantitative analysis of the fine particles (0.3 μm–2.4 μm)

Days of cultivation	Start	2	7	14	21	28
Sample size (*n*)	14	14	15	12	14	15

ANOVA showed no significant difference of average numbers of particles over the duration of the laboratory cultivation (*p* = 0.63 >> 0.05; F = 2.28 >> 0.7).

ANOVA showed a significant difference between the mean values over the duration of the cultivation (*p = 0.011* >> 0.05; F = 2.33 < 3.21).

The basic load during the test in the laboratory showed the values in Table 5:

**Table 5:** Values of basic load (coarse and fine fraction)

Mean coarse particles	1.5
SEM	0.4
Sample size (*n*)	46
Mean fine particles	5.8
SEM	1.2
Sample size (*n*)	24

## Discussion

The method showed both the properties of the moss leaf surfaces and the structure and size of the fine dust particles.

The SEM images were produced using dry moss leaves that were not turgescent. The possibility of working with liquid nitrogen or performing critical freezing drying was not available. The cell walls are therefore shown arched on the SEM micrographs of *B. rutabulum*, *H. cupressiforme*, and *C. purpureus*, and the originally water-filled cell lumina have collapsed. In *R. canescens* and *S. convolutum*, the papillae are much closer together than in the turgescent state (see Figs. 8, 9, 10, 11, and 12).

The selection procedure revealed that due to its growth habit, size, and shape of the leaflets, *B. rutabulum* was best suited as a model organism for the method presented here. Three of the five species of moss investigated (*C. purpureus*, *S. convolutum*, *R. canescens*) showed moderate loads of particulate matter. In two of these species (*S. convolutum*, *R. canescens*), the fine particles are difficult to detect between the papillae, making the assessment more complicated. The smooth leaflets of *H. cupressiforme* were easy to examine for fine particles, but the preparation for examination is difficult because the leaflets are hollow and much smaller than those of *B. rutabulum*. The leaflets of *B. rutabulum* are large and flat, with a smooth surface that was easy to examine under a SEM. The surface properties of *B. rutabulum* made it possible to carry out a quantitative analysis of the fine dust particles on the moss surface using the method presented here. It was not possible to do the quantitative analysis of particles on the surfaces for the other four moss species with the same quality for the reasons mentioned above. Therefore, no comparative studies on the occurrence of fine dust particles on the leaflet surfaces could be carried out in this work.

The SEM that was used only guaranteed a good resolution up to 3000-fold magnification. At this magnification, particles of up to 0.3 μm could still be reliably identified as particulate matter. Even with microscopes that allow a higher magnification, with a particle size of less than 0.1 μm, possible particulate matter cannot be clearly distinguished from cell wall formations (Nebel, unpublished).

Aboal et al. (2011) showed that particles on the moss surface of *Pseudoscleropodium purum* cannot be washed off even by soaking and shaking with water. The irrigation of the moss culture in the present work was performed with fine nebulization. Therefore, a possible effect of washing off the particles by irrigation was not investigated.

No comparative study on particulate matter deposits on different moss species with smooth or papillate surfaces has been published to date. The assumption that papillae, which increase the surface area and structural strength of the leaf surface, would promote the uptake of particulate matter could not be confirmed within the scope of this study.

The studies conducted by Di Palma et al. (2017) and by Tretiach et al. (2011) do not address the counting and degradation of particulate matter on living mosses. Other publications include SEM micrographs of individual moss leaves, but provide no quantitative analyses (Fabian et al. 2011; Hofman et al. 2017; Weinbruch 2010).

Manual counting, with the aid of the image processing program, reduces errors in the system and increases the reliability of subjective counting.

The SEM images were taken in the area of the leaf blade (see Fig. 4), because the cells are relatively homogeneous in this area (Smith 2004). Peripheral influences on the conditions for deposition of particles are least expected here. The same procedure was used as for species identification. The leaf blade is also used to ensure better comparability and clear expression of the features characteristic of the species.

The decrease in the number of particles on the moss surface of *B. rutabulum* may have several causes. Since the moss culture was kept permanently moist, a biofilm formed on the leaf surfaces. The fine dust particles could be broken down by physical-chemical processes of the microorganisms in the biofilm (Bai et al. 2021; Frahm 2001). Because mosses feed from fine dust on their leaf surface, particles may also have been internalized and metabolically processed inside the cell (Canivet et al. 2014). Under lab conditions, the moss plants were largely isolated from other nutrient sources. Mosses have few storage compartments; they are poikilohydry and cannot create consistent conditions (Frahm 2001). The close-meshed photo documentation showed an increase in biomass and proved metabolic activity for the duration of the study (see Fig. 7).

Based on the data collected, it cannot be answered whether only one or both hypotheses are correct.

- The quantitative analysis of the fine particles (2.5 μm–0.3 μm) showed a significant decrease (*p* = 0.011 >> 0.05; F = 2.33 < 3.21) of 16.9 ± 2.1 particles to 8.6 ± 2.4 particles in the course of the investigations.
- The quantitative evaluation of the coarse particles showed no significant decrease. The initial value was 4.0 ± 0.7 particles, and the basic exposure to coarse particles in a laboratory environment of 1.5 ± 0.4 particles is only slightly below the initial value.

Fine dust concentrations can locally fluctuate very much and depend, among other things, on the weather and microclimate. Further investigations with higher initial numbers are necessary here in order to be able to make a qualified statement about the decrease in coarse particles under laboratory conditions.

The investigations were carried out on living mosses. Dead mosses are also able to absorb substances, as the work of González et al. (2016) has shown, in this case, heavy metal compounds. To date, no experiments have been done on particulate matter uptake and reduction by dead mosses.

The present work shows a possibility to quantify the actual number of particles divided into two size categories on moss surfaces.

A weak point of the method used here is the determination of the lower limit of the structures that can be clearly identified as particulate matter particles. Below this limit, it is only partially possible to clearly assign structures on the moss surface. The evaluation of the fine particles is also particularly time-consuming and requires a large sample size.

The work carried out enables the collection of data and observations that are not possible with the moss bag monitoring method (Ares et al. 2012).

## Conclusion

Reliable data is required to put the discussion about particulate matter on a factual level. This in turn requires various measuring methods. The more reliable measurement data is available, the more precisely the fine dust problems of many urban areas can be analyzed. This also includes taking into account data on the number of particles. This is the basis for the development of solution concepts for the reduction of particulate matter at the urban hot spots. Mosses can make a contribution here due to their physiological properties.

A selection procedure makes it possible to find further moss species that are suitable for a quantitative analysis of the particulate matter load on their leaves with the method presented here. The evaluation criteria for the suitability of moss species are preparation of the moss leaflets for microscopy, visibility, and occurrence of the particles and the most important point the properties of the moss surface (see Tables 1 and 2).

## Data Availability

All authors assure that all data and materials as well as software application support their published claims and comply with field standards.
